# *Agave americana:* Characteristics and Potential Breeding Priorities

**DOI:** 10.3390/plants11172305

**Published:** 2022-09-02

**Authors:** Sarah C. Davis

**Affiliations:** Voinovich School of Leadership and Public Service, Ohio University, Athens, OH 45701, USA; daviss6@ohio.edu; Tel.: +1-740-597-1459

**Keywords:** drought, fiber, bioethanol, climate resilient crop, water use efficiency, Crassulacean Acid Metabolism

## Abstract

*Agave americana* L. is one of many *Agave* species that could be developed for the production of valuable agricultural products. Although all species in this genus use Crassulacean Acid Metabolism (CAM) and most have drought and heat tolerance, *Agave americana* also has the combined traits of high yield and cold tolerance. This review highlights key characteristics of *Agave americana* that make it an exceptional novel crop for fiber, sweeteners, bioproducts, and bioethanol with resilient traits for changing climate conditions. Then, it proposes potential directions for breeding that will support production in semi-arid climates. With selection and breeding, yields of 16 Mg ha^−1^ y^−1^ may be achieved. Current field observations, with no crop improvement, indicate ~9 Mg ha^−1^ is the maximum yield, and in arid regions, a yield of ~3 Mg ha^−1^ y^−1^ is observed. It may be beneficial to breed for a shorter time to flowering, as has been successful for *Agave tequilana* Weber var. azul, so that further breeding goals are achievable in a decadal timespan. Specific trait selection during breeding will depend on whether fiber or sugar yields are the desired products at a given location. Even without breeding, varieties of *Agave americana* are climate resilient alternatives for some current commodity crops.

## 1. Introduction

Recent crop research aims to optimize crop tolerance to environmental stress, but some plant species already have tolerances ideal for extremely variable growing conditions. For instance, some plants that use Crassulacean Acid Metabolism (CAM) persist through prolonged drought periods in addition to extreme heat [1,2], and are likely to be important germplasm resources for developing climate resilient crops in the future. In the case of *Agave americana* L., an obligate CAM plant, very little breeding may be required to produce valued products in regions with frequent droughts and increasing temperatures.

The CAM process is distinct from other photosynthetic strategies (i.e., C3 and C4), because plants with CAM assimilate carbon dioxide (CO_2_) at night instead of during the day; this conserves water in plant tissues because cooler temperatures at night lead to lower evapotranspiration than would otherwise occur during the day [1,2,3,4]. The carbon assimilated overnight is stored as an acid in large vacuoles [3]. The stomata, which are sunken on many succulent CAM plants (e.g., *Agave* spp.) to provide an extra boundary layer of air that minimizes the loss of water, stay closed during daytime hours while previously assimilated carbon is decarboxylated inside the leaves [3]. Photon energy harnessed during the day for the light reactions in chloroplasts generates energetic molecules that catalyze the Calvin cycle, whereby the CO_2_ (generated from the decarboxylation of carbon stored overnight) is then fixed by the enzyme ribulose bisphosphate carboxylase (RUBISCO) into carbohydrates [3]. The whole process of photosynthetic fixation of atmospheric carbon requires 24 h to complete in CAM plants [3].

Physiological adaptations associated with CAM result in enormous advantages for water use efficiency (WUE) relative to C3 and C4 plants [1,2,5]. The WUE of some CAM species can be 10–20 times that of C3 species and 2–5 times that of C4 species [5]. The result is that CAM plants can grow in regions where many plants cannot [1], such as in deserts and semi-arid grasslands.

There are over 200 species in the *Agave* genus [6,7] and all are obligate CAM plants, with most exhibiting high water use efficiency and extreme heat tolerance [8,9]. Still, few achieve the productivity levels of *A. americana* while also tolerating cold temperatures of as little as −9 °C [6]. The exceptional cold tolerance of this species means the potential growing region for *A. americana* is broader than other *Agave* species. Although currently grown primarily as an ornamental [6], *A. americana* has potential as a crop and can be the source of many different useful products [1,6]. This paper will review specific characteristics, geographic distribution, potential growing regions, uses for *A. americana,* and important issues for breeding.

## 2. Characteristics of *Agave americana* and Distinction from Other Species

Drought and temperature tolerances for *A. americana* were resolved in the early 1980s [10,11,12], but field observations of an *A. americana* crop were reported more recently [13]. As with many CAM plants, *A. americana* can survive prolonged periods of drought and make use of water whenever it is available. *A. americana* can survive with as little as 200 mm of annual rainfall that is not evenly distributed over the year [13], but average annual rainfall in its native growing range is 600–800 mm [6]. Some regions within the native range have a distinct rainy season, and dry conditions persist throughout much of the year [6,13].

Recent models estimate that a crop yield of 16 Mg ha^−1^ y^−1^ is possible with *A. americana* and that the growing region for this species is likely to expand with climate change, especially where warming occurs in higher latitudes [14]. These theoretical estimates of crop yields are grounded in physiological responses to light, temperature, and water inputs [15]. Based on current information, photosynthesis reaches a maximum rate at light levels >1000 µmol photons m^−2^ s^−1^, the optimum nighttime temperatures ranges from 10 to 30 °C, and optimal water inputs are 552 mm y^−1^ [15]. While nutrient additions would be required, and the optimum fertilization rates are not yet resolved, the resilience of this species in regions with drought and extreme heat is distinct from more common commodity crops (e.g., maize, soybean, rice, wheat), some of which will have contracted growing regions as climate change progresses [16].

Few species have the combined traits of high yield, cold tolerance, and drought tolerance that is found in *A. americana,* with the possible exception of *A. salmiana*, which reportedly yields 10–42 Mg ha y^−1^ in Mexico and has cold tolerance [2,17]. Temperature tolerances of *A. americana* are quite extreme relative to most other *Agave* used as agricultural crops (Table 1) [18,19,20,21,22]. Optimal nighttime temperatures are approximately 15 °C, but the plants photosynthesize with nighttime temperatures as low as −3 °C and will not suffer tissue damage until temperatures drop below −9 °C [22]. There are other *Agave* species with cold tolerance (e.g., *A. lechuguilla*, *A. neomexicana, A. parryi*, *A. utahensis*) [6,18,22], but these are not produced commercially and most of these have much slower growth than *A. americana*.

The heat tolerance of *A. americana* is also remarkable; this species can acclimate to daytime temperatures up to 63 °C and maintain maximum photosynthetic rates at daytime temperatures up to approximately 45 °C [12]. Photosynthesis remains active with nighttime temperatures up to 38 °C [11]. The wide range of temperatures at which this species can maintain optimal physiology is a distinct trait of interest for climate resilience and potentially for future breeding.

The optimal light environment for *A. americana* is ~1000 umol m^−2^ s^−1^ [15], so that shading from the leaves of individuals more densely planted is unlikely to limit growth. Therefore, plant spacing may be narrower than prior field trials. Each plant initially established in a crop (mother plants) will produce an average of five offset plants (pups) annually [13], providing an opportunity to propagate cohorts of plants for future harvests and potentially supporting sustainable management. To date, little work has been done to develop agronomic practices for the commercial production of *A. americana* [13].

*Agave americana* is monocarpic and can grow for several decades before maturing to the flowering stage. Although the plants could potentially be grown for more than ten years before harvesting, and a partial harvest of leaves would be possible during this time, there is a specialist weevil (agave snout weevil, *Scyphophorus acupunctatus*) that usually damages the plant before it reaches maturity [18]. Genetic variation is evident among plants in the wild and those available from nurseries, but clonal reproduction would likely be the most feasible strategy for long-term crop management due to the extended time to flowering and the increased risk from the snout weevil as the plant matures. In crop production, plants would most likely be harvested before flowering occurs.

In the first field trial of an *A. americana* crop in the southwestern United States, biomass production was maintained at a moderate level with only 300 mm of annual water input [13]. Biomass peaked at 9.3 Mg ha^−1^ y^−1^ with 530 mm of annual water input, and similar biomass resulted in experimental plots treated with greater water input [13]. The primary limitation on growth in this field trial was the agave snout weevil, which penetrates the core of mature plants when they reach a certain size. Greater water input resulted in faster growth, but the weevils killed the larger plants and limited the maximum growth that could be achieved at a plot level. The biomass production, even after the impact of the weevil, is similar to that of maize crops, but the water input required is much lower [1,13].

## 3. Geographical Distribution

*Agave americana* originates from a region that includes northern Mexico and the southwestern US, but it is now found throughout the world [6]. Spanish and Portuguese colonizers of Mesoamerica introduced *A. americana* to the Azores and the Canary Islands, and the species was well established in the Mediterranean by the 1700s [6]. It persists in the coastal Mediterranean today and has spread throughout six continents [23]. According to survey data collected by CABI, *A. americana* is considered invasive in Portugal, Spain, Italy, Greece, Ethiopia, Kenya, Uganda, Tanzania, Malawi, South Africa, Namibia, Japan, Australia, and New Zealand [23]. Despite the invasive categorization, there are places in Australia where *Agave* plantations of the last century did not lead to widespread populations in the wild. *Agave* spp. are considered naturalized in many more countries than those where they are labeled invasive, and found throughout South America, Africa, Europe, and Asia [23].

There are several subspecies and varieties of *A. americana*, including ssp. *protoamericana* Gentry, var. *expansa* (Jacobi) Gentry, and var. *oaxacensis* Gentry found in the US and Mexico [6,24]. Ornamental varieties include var. *marginata* Trel., var. *mediopicta* Trel., and var. *striata* Tel., all of which are found across much of the current range of *A. americana* [6]. Physiological variation is evident across these varieties, and crops would exhibit different traits if these varieties were incorporated into field cultivation [6,13].

## 4. Potential for Scaling Production

**Figure 1:**
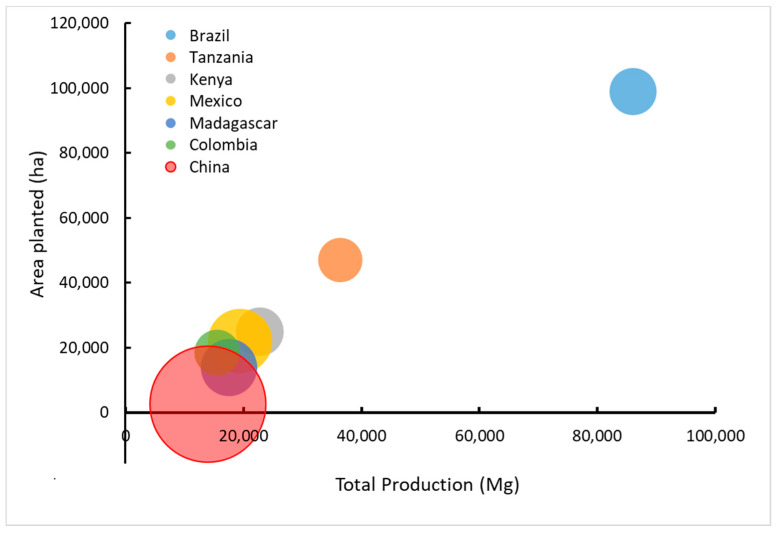
Countries with the greatest production of *Agave* fiber crops in relation to the amount of area harvested. The size of the circles corresponds to the yield per unit area of crops. Data reflect production in 2020 as reported by FAOSTAT [31].

**Figure 2:**
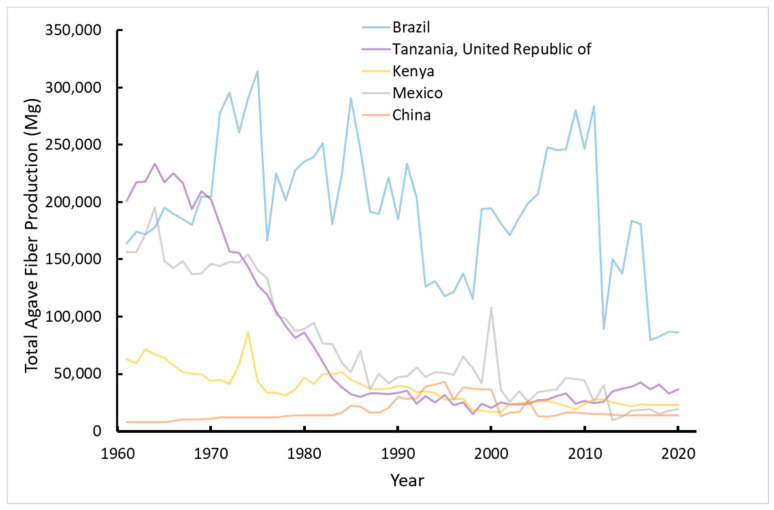
Production in Mg of fibers from four countries with the highest production over time and China, a recent top producer. Data source: FAOSTAT [31].

**Figure 3:**
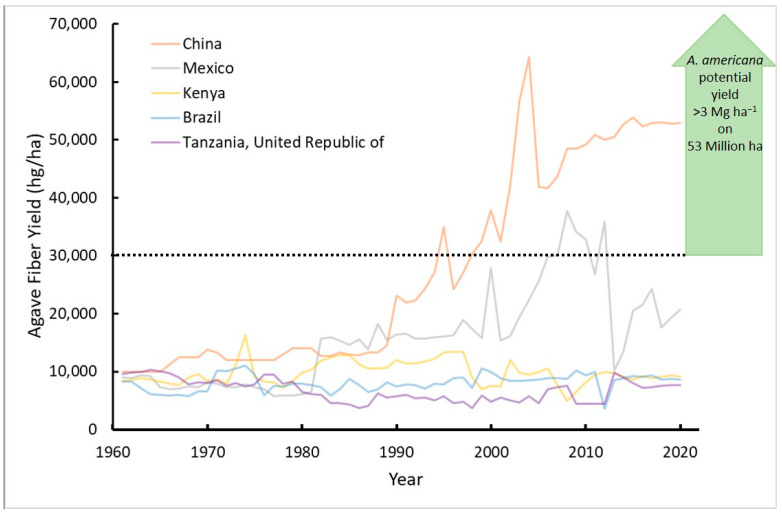
Yield in hg ha^−1^ of fibers from four countries with the highest production over time and China, a growing producer in recent years. The green arrow indicates theoretical production of *Agave americana.* Data reflect production as reported by FAOSTAT [31]. Potential yield of *A. americana* is based on modeled estimates [14].

Some species in the *Agave* genus have been cultivated commercially for fiber and beverages in Central and South America, Africa, and Asia [25,26]. *Agave tequilana* Weber var. azul, for example, has been bred to have a shorter lifecycle than other varieties, so the plants can be harvested in six to eight years for the high sugar content that becomes concentrated in the stems (also known as piñas or cabezas) just before flowering [27]. Others, such as *Agave fourcroydes* Lemaire and *Agave sisalana* Perrine, have been bred for their long straight leaves with high quality fibers [28,29,30]. The four nations that produced the most agave fiber in 2020, and for many years before, include Brazil, Tanzania, Kenya, and Mexico [31] (Figure 1); however, the amount produced has declined substantially since the peak of global production in the 1960s (Figure 2). Brazil has maintained agave fiber production over the last six decades, albeit with large year-to-year variations, but China reports the greatest yield per unit land area relative to all countries over the last 30 years (Figure 3) and was ranked seventh in total production in 2020 (Figure 1).

**Figure 4:**
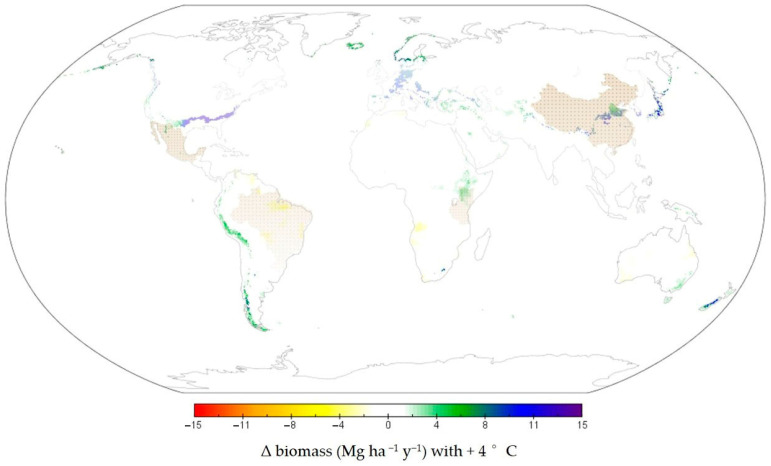
Five nations with greatest production of *Agave* crops for fiber (dotted brown) and expected change in potential biomass production of *Agave americana* with climate change as predicted with +4 °C warming (modified from [14]).

Despite the decline in *Agave* fiber production worldwide, there is a surging interest in bioproducts and textiles from natural fibers due to the realized risk of petroleum-based materials (e.g., [32]). *A. americana* could potentially be bred for fiber and could fill the gap in production left by declining fiber crops (Figure 3 and Figure 4). With high yield and extreme environmental tolerance, *A. americana* might require less land than other species, while also embodying a lower risk of crop loss due to unpredictable climatic events [14]. Globally, the area of *Agave* fiber crops has declined by 779,833 ha since 1964 [31], suggesting that 2.3 to 7.0 million metric tons of biomass from *A. americana* could be produced on land that was previously used for *Agave* fiber crops.

The commercial species typically used for fermented beverages (e.g., *Agave tequilana*) and fiber (e.g., *Agave fourcroydes*, *Agave sisalana*) do not have the cold tolerance of *A. americana* [6,21,22,29,33]. As a result, commercial agriculture for *Agave*, to make tequila/mescal or textiles/rope, has thus far been restricted to tropical and subtropical latitudes [34]. Recent analysis indicates that *A. americana* can be grown as a crop in northerly latitudes, and there is evidence of pre-Columbian cultivation at a large scale across the southwestern US [14].

With current climatic conditions, *A. americana* could be grown in some of the driest conditions of the desert southwest in the US. However, with 4 °C of global warming (relative to 2005), the growing region could also extend up the west coast as far as the northwest territories and into coastal Canada, and from the Mediterranean to Denmark (Figure 4) [14]. In future warming scenarios predicted by the recent Coupled Climate Intercomparison Project Phase 5 (CMIP5), even in regions with extreme drought expected (in parts of Australia, Africa, and South America), crops of *A. americana* could still be cultivated [14]. The expansive potential growing region for *A. americana* underscores the extraordinary physiological adaptations of this species.

Economic evaluations of the agricultural potential for *Agave* in the US indicate that labor costs for managing this crop hinder the economic viability of large-scale production [35]. Management practices employed for *Agave* production in other parts of the world involve skilled manual labor with specialized tools for slicing off the leaves of an *Agave* plant at the time of harvest [35]. In order for commercial production to be profitable in the US, for example, some mechanization would be required [35].

## 5. Traditional Uses and Cultivation

*Agave americana* is primarily used as an ornamental in xeriscapes, but it has a long history of traditional uses and has been evaluated recently for other valuable products [36]. It can be a source of sugar and nutrients, but also has medicinal uses as anti-inflammatory, antioxidant, and anticancer agents [36,37,38,39,40]. Traditional uses also include the production of pulque (a fermented beverage) and fibers for textiles similar to henequen or sisal [36]. There are currently no known commercial plantations of *A. americana*, although close relatives have been cultivated commercially as described in the previous section.

There are many examples of *Agave* cultivation that are not commercially scaled; subsistence farmers have historically used wild plants that grow in forest and grassland ecosystems [26]. Semi-domestication is also used in small-scale farming, where *Agave* plants are grown alongside other crops [26]. Small-scale management allows for maintaining different age classes of *Agave*, with only the mature plants harvested in a given year. These plants can be propagated both sexually and asexually, whereas commercially scaled production of *Agave* relies exclusively on asexual clonal propagation [28].

Over the last 15 years, there has been an increased interest in using *Agave* as an alternative biofuel and the plant tissue composition of *A. americana* is particularly well-suited to processing for bioethanol [25,41]. The plants have a high concentration of soluble sugars, a consequence of CAM physiology, that are easily fermented to ethanol, but they also have lower lignin content than many other advanced biofuel crops [41]. The low lignin content reduces the need for costly pretreatments and reduces waste production. Wastes from the processing of *Agave* for other products can also be co-digested in anaerobic digestion systems to make biogas [25,42].

In a direct comparison with advanced bioenergy feedstocks, *A. americana* plant tissue yielded greater carbohydrates for fermentation after enzymatic hydrolysis than perennial grasses [41]. The resulting amount of fuel that can be produced from *A. americana* per unit of water input required for cultivation is 49% greater than *Miscanthus* × *giganteus* Greef et deuter (giant miscanthus) and 325% greater than *Panicum virgatum* L. (switchgrass) [41]. This difference is remarkable considering that the total biomass yield of giant miscanthus is more than double that of *A. americana* and the biomass yield of the switchgrass is similar to *A. americana* [41].

## 6. Considerations for Breeding

Clonal propagation would be appropriate for large-scale agricultural production of *A. americana,* because two decades or more are required for the plants to reach the flowering stage [28,43]. Yet, there is a wide range of genetic variation evident in this species [42,44] and some selection for optimum traits would be desirable. Some of the traits depend on what product is most valued—e.g., fiber, bioethanol, medicinal extracts—and there would be some screening of these traits required before varieties were selected for production.

While drought, heat, and cold tolerance are consistently observed in *A. americana* varieties, there may be variations in growth or tissue composition under different environmental conditions that would warrant common garden experiments to guide selection. For example, there was substantial variation in both photosynthetic rates and growth rates among individual plants within treatment plots in Maricopa, Arizona [13]. The treatment effects associated with water input were larger than the variation among individuals within a plot, but the variation among individuals does suggest that breeding selection for desirable traits may improve yields [13].

**Figure 5:**
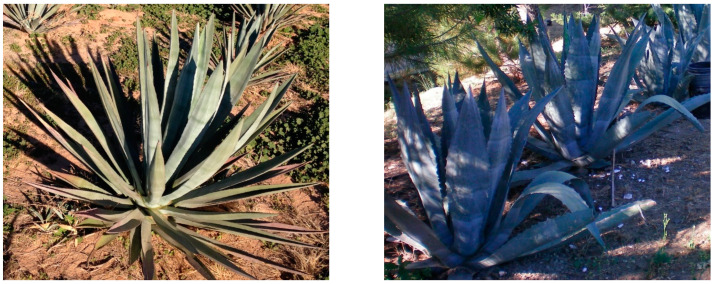
Straight leaves characteristic of *Agave fourcroydes* (**left**) and mixture of straight and curled leaves characteristic of *Agave americana* (**right**).

If *A. americana* is cultivated for fiber, breeding efforts should focus on selecting plants with optimal tissue composition and leaf shape. Some *A. americana* have long straight leaves, some have wider leaves with an exaggerated lanceolate shape, and some have leaves that are less rigid and curl like a ribbon (Figure 5) [6,42]. If fibers are harvested according to the traditional methods used for sisal or henequen, the long straight-leaved trait could be important for selection [25]. The shape of the leaves is less important if *A. americana* is bred for sweeteners or bioethanol [25,42].

The chemical composition of the leaves and stems would be more important than shape for sugar or ethanol products, where curling leaves that are less rigid may be associated with lower lignin content [25,41]. Prior analysis indicates that lower lignin content may allow for greater sugar and bioethanol yields after hydrolysis and fermentation, respectively [41]. Carbohydrate concentrations seem to remain relatively constant in *A. americana* [41], and leaves could be harvested annually for sugar products, but increased concentration of the soluble carbohydrates is likely to occur in stems just before the flowering stage [42]. Individuals that mature quickly would allow for more frequent harvest intervals.

If varieties are identified that are resistant to the agave snout weevil, this would be extremely important for crop breeding [13]. The snout weevil is the primary pest known to damage *A. americana* [18], an otherwise extremely resilient plant. In a field trial in the southwestern US with thousands of individual plants, all eventually succumbed to the snout weevil when they reached a certain size [13]. While this damage might at first seem to negate the viability of an *A. americana* crop, it is important for readers to note that the weevil only attacks plants when they reach a size greater than 30 kg, and the damage to stems that results actually makes the plants easier to harvest [13]. There are also pesticide applications that can be deployed to reduce the spread of the weevil. However, if resistance to the weevil was discovered in *Agave*, perhaps in relation to natural plant chemical defenses, that would be preferred by most current farmers as a guarantee of greater yields.

Some traits associated with *A. americana* may be desirable for breeding with other *Agave* species. Cold tolerance, for example, could be a trait of interest for species that are already produced commercially (e.g., *A. tequilana, A. fourcroydes, A. sisalana*) [1], so that the range for these crops can be expanded [13]. The species *Agave parryi* tolerates temperatures as low as −20 °C [12]. While *A. parryi* is lower yielding than many other *Agave* species [6], it offers an opportunity to breed even greater cold tolerance into *A. americana* while retaining traits that are ideal for agricultural products (e.g., fiber, sugar, etc.).

Breeding of *A. americana* has been very limited because of the long lifecycle of these plants [42,43], the large genome size [44], and the variation in ploidy levels [28,42]. The plants reproduce both sexually and asexually, and microclonal propagation provides practical reproductive tissue for agricultural production systems [42,43]. Transcriptome mining has proven useful for identifying gene expression levels associated with cellulose synthase [44] in *A. americana*, but there many other opportunities for probing the genes and transcripts that have not yet been realized [45]. As genetic resources become available in the coming years, new opportunities for breeding *A. americana* will follow.

## 7. Conclusions

There are many potential uses for *A. americana*, a high-yielding CAM plant closely related to species that are already grown commercially in warm climates for fiber, sugar, and tequila. With breeding, *A. americana* could be optimized for crop production in cooler climates at higher latitudes and could remain resilient in the event of drought and extreme heat. Pest resistance may be the most important consideration for breeding, but there is also an opportunity to breed for traits that are best suited for particular agriculture products. Some varieties are more ideal for fiber products, and others are more suited to yield sugar products. The value of *Agave* spp. is widely recognized in Central and South America already, but that value is currently unrealized in the United States and other countries seeking climate resilient crop alternatives.

## Figures and Tables

**Table 1:** Low temperature tolerance of *Agave americana* and other high-yielding *Agave* species in agricultural production (sources: [18,19,20,21,22]).

Species	Minimum Temperature Tolerance (°C)	Cold Acclimation
*Agave americana* L.	−10	yes
*Agave fourcroydes* Lem.	−2	no
*Agave salmiana* Otto ex Salm-Dyck	−6	yes
*Agave sisalona* Perrine	−7	no
*Agave tequilana* Weber var. azul	−4	no

## Data Availability

Data reported here are a review of previously published information, as cited in the text, and publicly available databases, including FAOSTAT (https://www.fao.org/faostat/ (accessed on 20 June 2022)).
